# Keeping the Myth Alive: Network Coordinators Facing the Challenges of Public Action in the Belgian Mental Health Sector

**DOI:** 10.5334/ijic.9050

**Published:** 2025-02-10

**Authors:** Coralie Darcis

**Affiliations:** 1 Johnson Shoyama Graduate School of Public Policy, University of Saskatchewan, Canada

**Keywords:** coordination work, governance, mental health, public policy, network model, myth

## Abstract

This thesis explored the issue of coordination in the Belgian mental health sector, focusing on “network coordinators” introduced through public policies setting up local networks to counter fragmentation. Using a qualitative and ethnographic methodology, the author examined the work of these new professionals from four perspectives: *instruments, practices, knowledge* and *experience*. Describing the contours of a promising but unachievable mandate, this thesis explained the *disillusionment* that they experience. Finally, it took a critical look at these coordination initiatives by showing the rareness of the “successful coordinator” and highlighting the limits of the *network model* as it is currently conceived.

## Summary

In this thesis we looked at the emergence of coordination professionals as one of the initiatives to coordinate actors within a system considered too fragmented, on the basis of a mandate issued by the public authorities. More specifically, our research was carried out in the field of mental health in Belgium, where three successive and complementary policies [1,2,3] were formulated to initiate a paradigm shift, from a hospital-centered, strongly institutionalized and segmented model toward a community-based, patient-centered and more integrated model [4]. These policies established inter-organisational and inter-sectoral networks and created several dozen ‘network coordinator’ positions responsible for supporting the implementation of these policies locally.

The thesis main research question was “How does the new position of network coordinator take shape in the Belgian mental health landscape and what does it tell us about how the implementation of mental health policies is governed?”. We structured the thesis around four articles in which we adopted four perspectives, addressing the network coordinator first as an *instrument of public action*, then as a *translator*, then as a *knowledge worker* and finally as a new professional group experiencing a form of *disillusionment*.

We opted for a qualitative, ethnographic research approach. Three main methods of data collection were used: (i) documentary analysis (e.g., of political and legal documents and documents produced by the actors during the implementation of the policies); (ii) semi-structured interviews (N = 68) with key stakeholders (i.e., network coordinators, network partners and policy makers and members of the administration); and direct observations (N = 96) of network coordinators in action and of the functioning of their networks. The focus group method was used on two additional occasions. We carried out a transversal thematic analysis of the collected data.

Despite the essential role delegated to these new workers in the launch of these policies, we showed that introducing the job of network coordinator in the mental health sector is not without its challenges. We pointed out the obstacles created by the ambiguous, complex and largely uncertain context for which this new role is intended and have observed that the boundaries of this new professional group (its mandate and license) [5] were still unclear and subject to negotiation. We showed that network coordinators lacked the professional legitimacy to carry out the mission entrusted to them, i.e., that of governing the implementation of a paradigm shift at local level.

As a rather weak actor within the system who strongly lacked the leverage to overcome structural obstacles, the coordinator tried to fulfil their mission by operating between the cracks of the varying constraints in the field structure. The thesis showed that, through the management of translation and knowledge creation processes [6,7,8,9] and by developing meta-knowledge [10], the coordinator was trying to create links within this new system, maintain the commitment of the partners and govern their network. And although it produced some results, such as softening certain organisational and sectoral boundaries and transforming professional practices, those remained marginal and far removed from the envisioned paradigm shift. Faced with the impossibility of carrying out their mission, numerous network coordinators experienced a form of disillusionment, leading some of them to experience burn out or to leave this initially promising job.

Using the notion of a ‘*myth of public action*’, we questioned more broadly the model of the mandated network as a solution to the problem of fragmentation as well as the modes of public action that characterise these new public policies. We identified some of the *false promises* that lied at the foundation of the myth and identified the role of the coordinator who, by encouraging these actors to take ownership of the myth, contributed directly to keeping it alive. This research questioned the need for a more profound change than the one allowed by the networks, an observation that has been emphasised for several decades by numerous field actors as well as by other researchers [11,12,13,14,15,16,17]. It highlighted the need to develop the structural conditions necessary for the implementation of the desired change and highlighted the need to clarify the status of network coordinators in the “local division of coordination labour” [18: 10]. Finally, it also emphasised the need to clarify the status of networks, especially at a time when these new structures are multiplying and sowing confusion in the system both for professionals [19] and for patients and their relatives.

This research highlighted, like other researchers before, the importance of this research subject. On the one hand the calls for coordination have become increasingly present over the last few decades, occupying the political agenda within the health sector as well as elsewhere. Indeed, a very large part of public action within the sector is based on the idea that better coordination is a key solution to what is seen as the *problem of fragmentation* and therefore integration of care. The promises associated with these policies and with the *imaginaries* [20] they deploy are significant. On the other hand however, research into these ‘attempts at coordination’ often reported mixed results and highlighted the many pitfalls faced by the actors involved in their implementation [21,22]. This research is a further illustration of this. For these reasons coordination is far from being a *simple solution* that could easily solve the problems arising from the increasing fragmentation of the sector.

It is therefore essential to deepen our understanding of the forms that coordination continues to take, in its mandated or spontaneous and inter- or intra-organisational forms, the issues that it raises, its achievements and pitfalls. More broadly it is also essential to keep questioning those forms of public action that fully rely on the supposedly performative power of certain ideas and *myths*. All of this will help us to question under what conditions these myths can be brought to life [23] and enable us to think of other *imaginaries* [20] alongside the “network ideology”.
